# Ethnobotanical study of wild edible fruits in eastern Bhutan

**DOI:** 10.1186/s13002-022-00526-8

**Published:** 2022-03-30

**Authors:** Pema Yangdon, Tetsuya Araki, Yen Yen Sally Rahayu, Kunzang Norbu

**Affiliations:** 1 Agriculture Research and Development Sub-Centre, 42-002 Khangma, Kanglung, Trashigang, Bhutan; 2 Department of Global Agricultural Sciences, Graduate School of Agricultural and Life Sciences, The University of Tokyo, 1-1-1 Yayoi Bunkyo, Ward Tokyo, 113-8657 Japan

**Keywords:** Wild edible fruits (WEF), Ethnobotany, Indigenous knowledge, Eastern Bhutan, Consumption

## Abstract

**Background:**

In the past, wild edible fruits (WEFs) were a significant source of food and nutrition in Bhutan. These nutrient-rich species can enhance food security and alleviate poverty in Bhutan. However, recent developments like the introduction of improved fruit varieties, changes in dietary choices, and infrastructure development are expected to influence indigenous knowledge and consumption of WEFs. We aimed to document the species diversity of WEFs and their uses in eastern Bhutan and examine how the knowledge and consumption of WEFs vary with socio-demographic factors.

**Methods:**

A total of 97 households in two districts were selected to participate in the survey. A semi-structured questionnaire was used to interview a selected adult from each household. Comparative analysis of indigenous knowledge and consumption of WEFs among the socio-demographic factors was performed using one-way ANOVA and a Chi-square test on R software.

**Results:**

The present study reported 52 species of WEFs belonging to 35 families. The prevalence of WEF consumption was found to be 42%. WEF consumption differed significantly between districts, age groups, and indigenous knowledge levels. Similarly, indigenous knowledge of WEFs was significantly associated with districts and age groups.

**Conclusions:**

Eastern Bhutan has a rich diversity of WEFs, but their consumption has been decreasing. Recent agricultural and infrastructure developments may have impacted the consumption and indigenous knowledge of WEFs in this region. Thus, domestication and agro-processing of WEFs should become a major focus in Bhutan to utilize their nutritional value and potential economic benefits to enhance food security in the country. Additionally, incorporating WEF-related knowledge in the school curriculum is essential to educate younger generations on WEFs.

## Introduction

Wild edible fruits (WEFs) refer to edible fruit species which are not cultivated but are collected from their natural habitats [1]. WEFs are mainly consumed during off-season periods of cultivated fruits and vegetables, predominated by food shortage [2, 3]. Even though agricultural communities rely mostly on improved cultivated varieties due to their nutritional value, health benefits, and higher productivity, the habit of consuming wild foods has not been entirely abandoned [4, 5]. Moreover, the world population is expected to surpass 9 billion by 2050, boosting global food demand by 50% compared to 2013 [6]. Thus, to meet the global food demand, the domestication of other food-producing species and intensifying the use of underutilized and neglected species, including wild food resources, may become necessary [7]. Wild food resources comprise a variety of edibles, including WEF, vegetables, mushrooms, orchids, canes, and herbal plants; and WEFs contribute the most to the total number of wild edible resources [8]. These nutrient-dense fruits have been discovered to be good sources of vitamins, minerals, and antioxidants [9–12]. As a result, in most of the developing countries, WEFs constitute a vital source of food, healthcare, and material subsistence and are linked to human survival [13, 14].

Landlocked Bhutan, widely regarded as the sole carbon-negative country in Asia and sandwiched between China and India, has an overall forest cover of 71%, with 51.44% covered by protected areas and biological corridors [15]. Bhutan is, thus, one of the world's biodiversity hotspots, housing over 11,000 species [16]. The dense forest and different agro-ecological zones in the country favor the growth of a wide range of wild edible plants. These species are excellent sources of food, medicine, fuel, animal feed, and timber and have various household and ritual applications. Similarly, numerous WEFs are employed in oil extraction, dyeing, and traditional medicine. As a result, it has significantly contributed to the food and nutritional well-being of rural Bhutan [17]. In contrast, another study reported that one out of three Bhutanese suffered from food insecurity, with nearly 30% of the population facing malnourishment and related health issues such as stunting [18]. Additionally, the Poverty Assessment and Analysis Report 2017 estimated that 8.1% of the Bhutanese population was under the national poverty line of Nu 2195.95 income per person per month, with a significantly higher poverty rate in rural areas. Hence, with its high nutrient content and potential for income generation through value addition, WEF species can considerably contribute to food security and poverty alleviation in remote areas of Bhutan.

However, the government's push for commercialization and the promotion of high-yielding cultivars in recent decades threatens to erode traditional WEF use in Bhutan [17]. Moreover, the reliance on wild edibles is likely to diminish over time because of the easy accessibility of improved varieties [19, 20], the decline in species diversity owing to habitat destruction through deforestation [21, 22], and infrastructure development [23]. As a result, indigenous knowledge and the consumption of WEFs are rapidly declining among the younger generations. The extinction of indigenous knowledge is also found to be linked to the reduction of plant diversity [24]. With the increasing erosion of indigenous knowledge on WEFs and increasing reliance on improved fruit varieties, there is a risk of complete substitution of wild fruits with imported fruit types, resulting in the disruption of the coexistence of people and forest, and loss of traditional knowledge sooner.

Thus, it is crucial to document the diversity of wild species and their indigenous potential for sustainable management of wild resources [25] before the extinction of indigenous species and their traditional knowledge. Although few previous studies have been conducted on wild vegetables, non-wood forest products, and medicinal herbs [26–29] in southern, southwestern, and central parts of the country, no study has focused particularly on WEFs in eastern Bhutan. Furthermore, these studies have focused intently on listing out the wild edible plants and their uses, while a comparative analysis on indigenous knowledge and the consumption of WEFs has not been conducted. We hypothesized that the consumption of and indigenous knowledge about WEFs are decreasing among the younger generations of Bhutan. Eastern Bhutan has the largest land area and the greatest number of rural households in the country [30]. Additionally, more than 70% of the land is under forest cover, making the region ideal for conducting an ethnobotanical study related to WEFs. Hence, in this study, we aimed to document the species diversity and ethnobotanical uses, compare indigenous knowledge and consumption of WEFs among socio-demographic factors.

## Materials and methods

### Study area

**Fig. 1:**
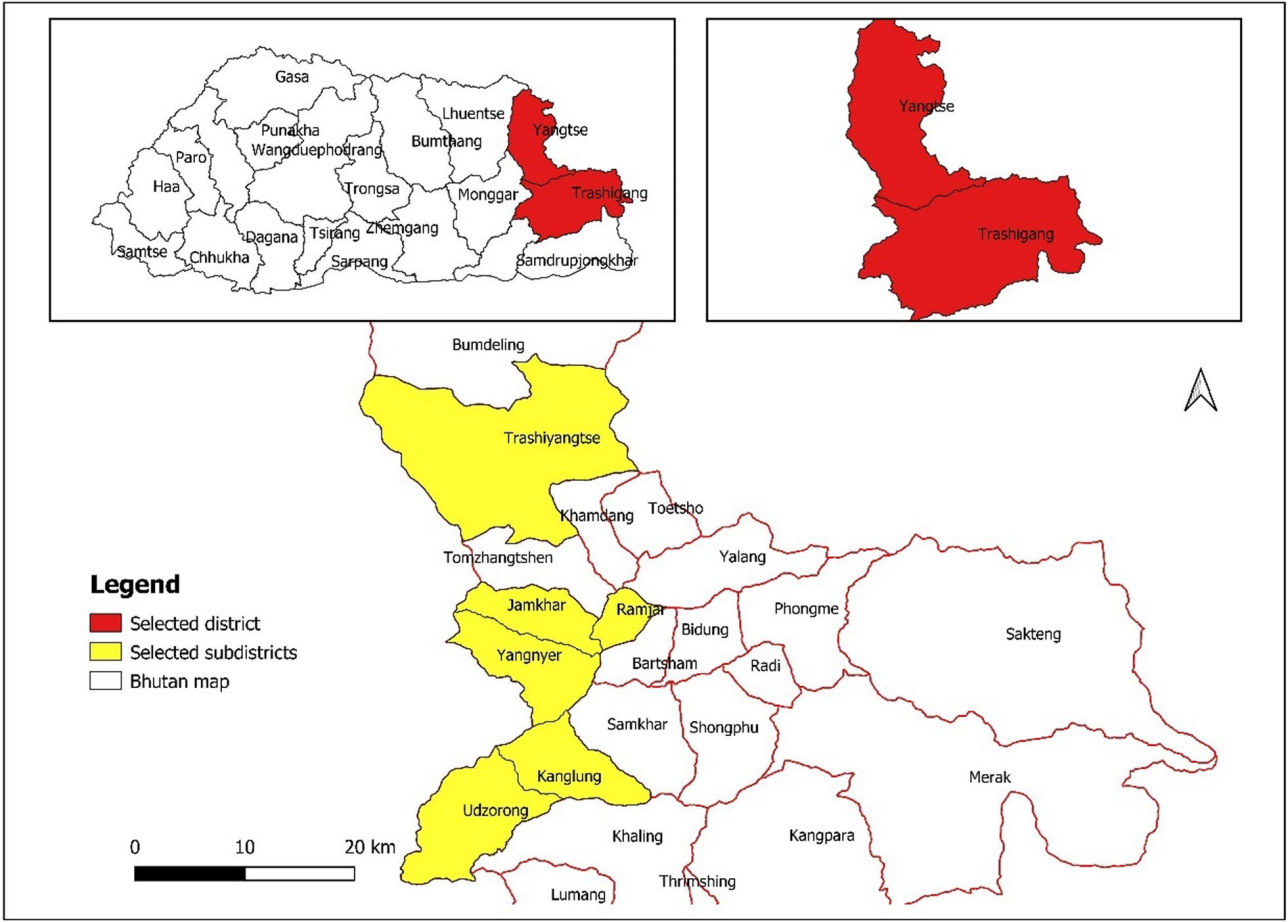
Study area map showing two districts and six sub-districts

The eastern part of Bhutan is the largest region in Bhutan, comprising six Dzongkhags (districts). The region has more than 70% of the land under forest cover [15]. It is considered to be less developed, with a higher poverty rate than the rest of the country [31]. Moreover, the region has the greatest number of rural households dependent on agriculture and is closely associated with nature and forests. This study was conducted in the Trashigang and Trashiyangtse Dzongkhag, located 501 km and 533 km, respectively, toward the east of the capital city of Bhutan, Thimphu (Fig. 1).

The survey was conducted in six Gewogs (sub-districts): Udzorong, Kanglung, and Yangneer in Trashigang; and Yangtse, Ramjar, and Jamkhar in Trashiyangtse Dzongkhag. These Gewogs have been selected based on their varying elevations and rural households. Both Dzongkhags have a rich forest cover, distinct land size, elevation, and household number (Table 1). Trashigang has the largest land area and households among all Dzongkhags in the eastern region, while Trashiyangtse is the second smallest. Almost all the Gewogs in both the Dzongkhags are connected to the Dzongkhag administration by farm roads, and each Gewog has at least one Basic Health Unit (BHU) for medication.

**Table 1:** Profiles of the study area

	Trashigang Dzongkhag	Trashiyangtse Dzongkhag
Total area (km^2^)	3060	1437.02
Number of households	9147	3697
Forest cover (%)	73	70
Distance from the capital city (km)	501	533
Total geogs	15	8
Elevation (m)	550–4600	600–3200
Mode of transport	Farm road	Farm road

The eastern region has a warm temperate climate in the northern part and a subtropical climate in the southern parts. Agriculture is the main source of income and livelihood for the rural populations. In 2010, the Japan International Cooperation Agency (JICA), in collaboration with the Ministry of Agriculture and Forests, initiated the Horticulture Research and Development Project (HRDP) to promote horticulture as a source of income in the six eastern Dzongkhags, in which more than 40 varieties of improved fruits and vegetables were introduced [32, 33]. Hence, farmers cultivate many improved fruits like pear, peach, plum, persimmon, and kiwi for consumption and commercial purposes. Farmers sell their fruits and vegetables to the nearby market or along the highway. In addition to farming, livestock rearing is one of the main sources of livelihood in the region. Usually, farmers herd their cattle in the forest, during which they collect and consume WEFs.

### Data collection and analysis

Information on demographic characteristics, diversity of WEF species, and their associated ethnobotanical uses were collected using semi-structured interviews in 97 households: 54 in Trashigang and 43 in Trashiyangtse Dzongkhag. Most respondents were farmers and housewives in Dzongkhags, while seven respondents in Trashigang were local healers and lay monks.

The Agriculture Extension Officer of the Gewog and the village heads were informed before conducting the survey. Respondents were selected from six Gewogs of the two Dzongkhags. Questionnaires were prepared in the KoBo Toolbox.^1^ Data were collected between September and October 2021 in both the Dzongkhags. Specimen collection and accurate WEF plant identification were completed in December 2021. For this study, four Bhutanese interviewers were recruited and trained on the data collection process. Before starting the interview, the nature of the research and its purpose were explained to obtain oral consent from each respondent. Interviews were conducted in Bhutanese languages and transcribed in English by the first author assisted by the four interviewers. In this study, WEFs were defined as any edible fruits that are not domesticated by farmers and found in the forest or any uncultivated land.

The WEF plants cited by respondents were recorded by their vernacular names in the Bhutanese language. The plants or fruit samples available were collected and photographed through guided tours in the surrounding fields and nearby forests. Collected plants or fruit samples were identified by comparing their characteristics to the literature from the Flora of Bhutan [34]. Voucher specimens were prepared for those species that the authors could not identify. The vouchered specimens were identified by the officials at the Agriculture Research and Development Centre (ARDC), Wengkhar, and validated by the officials at the National Biodiversity Centre (NBC), Thimphu. The specimens were deposited at the National Herbarium in the NBC, Thimphu. The scientific names of the species were updated according to Kew’s database—Medicinal Plant Name Services [35] and the World Checklist of Selected Plant Families [36].

R software was used for data analysis to estimate the frequency measures. One-way ANOVA and Chi-square test were used to compare the indigenous knowledge and consumption of WEF, respectively, among the variables.

## Results

### Diversity and use pattern

The study area yielded a total of 52 WEF species belonging to 47 genera and 35 families, including 29 (54%) trees, 13 (26%) shrubs, 5 (10%) herbs, and 5 (10%) climbers. The family Rosaceae contributed the highest proportion of WEF species with five species, followed by Rutaceae and Lauraceae with four species each. Moraceae and Anacardiaceae contributed three species each, while Combretaceae and Myrtaceae contributed two species each and the remaining families contributed only one species each. Out of 1,258 citations, the most cited WEF was *Rubus ellipticus,* with 89 citations, followed by *Docynia indica* and *Juglans regia* with 86 and 83 citations, respectively. Among the 52 species, 26 were collected from the forest and 14 from the surrounding fields. Twelve species showed no habitat preference as they were collected from both habitat groups (Table 2).

**Table 2:** List of WEFs and their collection seasons, availability, and uses in eastern Bhutan

Family	Botanical names	Local name	Collection number	Number of citations		Collection season	Availability	Consumption mode and other uses	References
				Trashigang	Trashiyangtse				
Actinidiaceae	*Actinidia callosa* Lindl.^a^	Zhimpeykotong/Phangkulomsey	KN015	5	0	Oct–Nov	Mo	Fruits are consumed raw on ripe. Stems used for making ropes and seeds for raising rootstock	
Anacardiaceae	*Rhus chinensis* Mill^a^	Roptang sey	KN013	10	0	Jul–Aug	Ra	Fruits are consumed raw on ripe. Stems and trunks are used as firewood and fencing poles. To treat snakebite and stomachache	[48]
Anacardiaceae	*Mangifera sylvatica* Roxb.^b^	Amsey	KN014	4	5	Jul–Aug	Mo	Fruits are consumed raw and as pickles	[49]
Anacardiaceae	*Choerospondias axillaris* (Roxb.) B.L.Burtt & A.W.Hill^b^	Phrumchung sey	KN012	14	0	Jul–Aug	Ra	Fruits consumed raw and as pickle	[49]
Araceae	*Colocasia esculenta* (L.) Schott^b^	Bozong	KN016	1	1	Jan–Feb	Ra	Corms are boiled and consumed as snacks, curry, and soup. Leaves used to wrap dairy products and as a plate	[21, 23, 48, 49]
Bignoniaceae	*Oroxylum indicum* (L.) Kurz^b^	Namkaling	KN020	2	0	Aug–Sep	Ra	Flowers are consumed as a vegetable and used for religious purposes. To treat burns and wounds, relieve cough and gastritis	[48, 49]
Combretaceae	*Terminalia bellirica* (Gaertn.) Roxb.^a^	Baru	KN025	1	5	Nov–Feb	Ra	Dried fruits are chewed for medicinal purposes. Fruits used for traditional medicine and religious purpose, used for cough and sore throat, diarrhea, ingestion, asthma, constipation, dye extraction	[21, 23, 49]
Combretaceae	*Terminalia chebula* Retz.^a^	Aru	KN021	1	5	May–June	Ra	Dried fruits are chewed for medicinal purposes. Used for traditional medicine and religious purpose. To treat cough, asthma, diarrhea, and constipation	[23]
Cornaceae	*Cornus capitata *Wall^c^	Maminpa sey/poitse sey	PY001	42	20	Sep–Oct	Mo	Fruits are consumed raw	
Cucurbitaceae	*Coccinia grandis* (L.) Voigt^c^	Khakhari sey		2	0	June–July	Mo	Fruits are consumed as a vegetable	[21, 48]
Dioscoraceae	*Dioscorea bulbifera* L.^a^	Borang joktang	KN022	19	3	Oct–Nov	Ra	Aerial and underground tubers are consumed by boiling or roasting. Stems are used as rope for fencing	[21]
Ebenaceae	*Diospyros lotus* L.^b^	Amdrebu sey/ gundum	KN024	6	9	Sep–Oct	Mo	Fruits are dried and consumed. Seeds are used to raise rootstock	
Elaeagnaceae	*Elaeagnus latifolia* L.^a^	Dangmaling sey	PY002	44	27	Jul–Aug	Ab	Fruits are consumed raw or processed into wine	
Elaeocarpaceae	*Elaeocarpus lanceifolius* Roxb^a^	Gashathung sey	KN023	24	10	Jul–Sep	Ra	Fruits are consumed raw as a pickle. The wood is used as a construction material, to make tea boxes and charcoal	
Ericaceae	*Vaccinium retusum* (Griff.) Hook.f. ex C.B.Clarke^a^	Shakshingma sey	PY008	35	17	Oct–Nov	Mo	Fruits consumed raw on ripe. Used for religious purposes and to treat a skin problem	
Fabaceae	*Tamarindus indica* L.^a^	Tetari	KN026	5	0	Mar–April	Ra	Pods are consumed raw or as pickles used to relieve constipation, as an appetizer, and spice/condiment	[48]
Fabaceae	*Castanopsis indica* (Roxb. ex Lindl.) A.DC.^c^	Tsha tsha sey	KN030	18	9	Oct–Nov	Mo	Nuts are consumed raw, after boiling and roasting. The wood is used as firewood and furniture making	
Juglandaceae	*Juglans regia* L.^c^	Khesey	KN027	53	30	Sep–Oct	Ab	Nuts are consumed raw, wood for Furniture, nut cover as a yeast, flowers as tea leaves, and seeds as butter in butter tea	[23]
Lardizabalaceae	*Holboellia latifolia* Wall.^a^	Throkchang sey	KN029	27	9	Nov–Dec	Mo	Fruits consumed raw on ripe. Stems used as a rope for plowing and fencing	
Lauraceae	*Litsea cubeba* (Lour.) Pers.^c^	Neng	KN028	22	7	Jul–Aug	Ra	Fruits are consumed as salad, dried, and processed into powder. Used as a spice in the food	
Lauraceae	*Litsea glutinosa* (Lour.) C.B.Rob.^a^	Kherim sey	PY009	1	4	Sep–Aug	Ra	Consumed as a spice. Used for oil extraction. Used as a cooking oil	
Lauraceae	*Machilus edulis* King ex Hook. f.^b^	Goli	PY011	21	13	Sep–Aug	Ra	Fruits are consumed as a substitute for curry with rice. Wood is used for firewood, seeds used to raise rootstock	
Lauraceae	*Parasassafras confertiflora* (Meisn.) D.G.Long^*c*^	Singsi	KN031	16	2	Aug–Sep	Ra	Fruits are used for oil extraction, used as cooking oil, wound treatment, skin lotion	
Moraceae	*Ficus auriculata* Lour.^c^	Chongma sey	KN035	32	18	Jul–Aug	Ab	Fruits are consumed raw or as dried fruit. Leaves and stems as fodder, leaves are used to wrap dairy products. The latex used to treat wounds and cuts, roasted fruits for treating diarrhea and dysentery	[21, 23, 49]
Moraceae	*Ficus semicordata* Buch.Ham. ex Sm.^c^	Barchongma sey	KN033	17	21	Jul–Aug	Mo	Fruits are consumed raw on ripe. leaves and stems as fodder, fruit paste for fever and menstrual disorder	[21, 49]
Moraceae	*Morus serrata* Roxb.^b^	Shagongma sey	PY003	6	2	Jul–Aug	Ra	Fruits are consumed raw. leaves used as a fodder	
Musaceae	*Musa acuminata* Colla^b^	Laisey	KN032	7	8	June–July	Mo	Fruits are consumed raw and processed into chips. leaves to wrap dairy products, as fodder for cattle	
Myricaceae	*Myrica esculenta* Buch.-Ham. ex D.Don^a^	Tsutsu sey	PY004	50	8	Jul–Aug	Mo	Berries are consumed raw on ripe. Used for cough and cold, leaves as fodder	[21, 49]
Myrsinaceae	*Ardisia macrocarpa* Wall^a^	Thakchung sey	PY005	8	1	Dec–Jan	Ab	Fruits are consumed raw on ripe	[21]
Myrtaceae	*Psidium guajava* L^b^	Bebsey	KN034	7	5	Nov–Dec	Mo	Fruits are consumed raw on ripe. leaves paste for hair growth and dandruff. Leaves are used as a green tea	[49]
Myrtaceae	*Syzygium cumini* (L.) Skeels^a^	Mintse	PY006	5	0	Jun–July	Ra	Fruits are consumed raw on ripe. Used as firewood and fodder, to treat diabetes, diarrhea, dysentery	[21, 23, 49]
Nephrolepidaceae	*Nephrolepis cordifolia* (L.) C.Presl^*a*^	Ata khaw khaw	KN036	1	0	July–Aug	Ra	Underground nodules are consumed as a refreshment. Used to treat hypertension and diabetes	[21]
Passifloraceae	*Passiflora edulis* Sims^b^	Zargong	KN040	5	0	Aug–Sep	Mo	Fruits are consumed raw on ripe	[48]
Pinaceae	*Pinus roxburghii* Sarg.^a^	Tongphu shing	KN037	1	1	Nov–Dec	Mo	Seeds are consumed raw or after roasting. Wood as a construction material	
Punicaceae	*Punica granatum* L.^b^	Tshalem/Thalem	KN038	4	11	April–May	Mo	Fruits are consumed raw on ripe	[23]
Phyllanthaceae	*Phyllanthus emblica* L.^a^	Chorgen sey	KN039	11	25	Dec–Feb	Ab	Fruits are consumed raw or processed into a pickle, dried fruit, and wine. Used to treat hypertension, cough, and cold. Used for traditional medicine and religious purpose	[21, 23, 48, 49]
Rhamnaceae	*Zizyphus mauritiana* Lam.^a^	Khangaring	KN041	9	19	Feb–Mar	Ab	Fruits are consumed raw and processed into pickles and wine. for traditional medicine. Used for cuts and ulcers, ingestion, and fever	[21]
Rosaceae	*Chaenomeles lagenaria* (Loisel.) Koidz*.*^b^	Khomang	KN042	19	0	Sep–Oct	Mo	Consumed raw or processed into a pickle. Used as a dye. Used for treating cough and cold, to remove the rust	
Rosaceae	*Docynia indica* (Colebr. ex Wall.) Decne.^C^	Thungkakpa	PY010	49	37	Nov–Dec	Mo	Fruits are consumed raw, after boiling or roasting, and as dried fruit. Used as an appetizer and to cure diabetes	[49]
Rosaceae	*Fragaria nubicola* (Lindl. ex Hook.f.) Lacaita^a^	Sagong	KN043	34	21	June–July	Ab	Berries are consumed raw and processed into juice	
Rosaceae	*Pyrus pashia* Buch.-Ham. ex D.Don^c^	Letong	KN044	18	15	Oct–Nov	Mo	Fruits are consumed raw, like dried fruit, and processed into wine. Seeds used as a rootstock material	[23]
Rosaceae	*Rubus ellipticus* Sm.^c^	Sergong	PY007	49	40	May–June	Ab	Berries are consumed raw and processed into juice	[21, 23]
Rutaceae	*Zanthoxylum armatum* DC.^a^	Gee	KN045	33	22	Aug–Sep	Mo	Berries are used as a spice. Used to ward off evil spirits	[23]
Rutaceae	*Citrus* × *aurantiifolia* (Christm.) Swingle^b^	Kapur	KN050	4	16	Nov–Dec	Ra	Fruits are consumed raw as a salad, processed into a pickle. Used to remove dandruff	[23]
Rutaceae	*Citrus medica* L.^b^	Lumpang	KN048	7	13	Nov–Dec	Mo	Fruits are consumed raw and processed into juice and pickle. Used to relieve pain and inflammation, treat skin disorder and itching	[23]
Rutaceae	*Murraya koenigii* (L.) Spreng.^a^	Berkang sey/Lebi sey	KN046	3	6	July–Aug	Mo	Fruits are consumed raw. Used as a condiment in curry	[21, 23, 49]
Rubiaceae	*Catunaregam spinosa* (Thunb.) Tirveng.^a^	Ngyerthung sey	KN047	5	2	Nov–Dec	Ra	Fruits are consumed raw on ripe	
Sapotaceae	*Diploknema butyracea* (Roxb.) H.J.Lam^a^	Fin sey/Phinlung	KN049	15	7	June–July	Ra	Fruits are consumed raw on ripe. Used for oil extraction. Used as cooking oil, butter lamp, to treat a pimple, boils, burns, headache and rheumatism	[21]
Schisandraceae	*Illicium verum* Hook.f.^c^	Wunba tsinang	KN053	2	0	Sep–Oct	Ra	Seeds are consumed with betel leaf. Used as a spice in tea and curries	
Solanaceae	*Physalis peruviana* L.^c^	Pokpokma sey	KN050	2	0	June–July	Ra	Fruits are consumed raw or cooked as a vegetable. To treat diabetes and hypertension	
Symplocaceae	*Symplocos paniculata* Miq.^a^	Thulu sey/ Pangtse shing	KN052	1	0	Aug–Sep	Ra	Fruits are consumed raw on ripe. Used for oil extraction which is used as cooking oil. Leaves used as a dye	
Thymelaeaceae	*Daphne bholua* Buch.-Ham. ex D.Don^a^	Desho shing	KN051	0	7	May	Ra	Fruits are consumed raw on ripe. The bark is used for making paper and rope	

References—commonly listed species in Nepal, India, Laos, and Myanmar

Availability—Ab: Abundant; Mo: Moderate; Ra: Rare

Botanical names—^a^forest, ^b^Field surrounding, ^c^Both

**Fig. 2:**
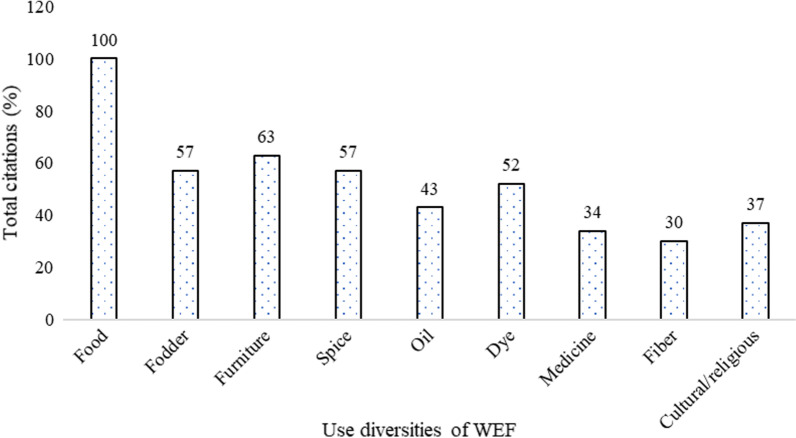
Total citations for WEF uses

We found that WEF species served various purposes for the rural people. Besides food, the species have multiple uses as medicine, spices, oil, dye, fiber, fodder for livestock, raw materials for furniture, and cultural/religious purposes. The most cited use was food, followed by their use as a raw material for furniture and construction, spices, fodder, dye, and the other uses had fewer citations (Fig. 2). The number of citations for WEF uses did not differ between the two Dzongkhags except for higher medicinal use in Trashigang Dzongkhag (*X*^2^ = 3.836, *df* = 1, *p* < 0.05). In addition to the fruits, the respondents also used other plant parts such as seeds, underground parts, and flowers as food. The proportion of species consumed both in raw and cooked/processed forms was 43%, whereas 37% were consumed raw and 20% were consumed in cooked/processed forms.

### WEF consumption

The survey result showed that only 42% of the respondents collected and consumed WEFs within the last twelve months. The proportion of the respondents who consumed WEFs within the last twelve months was compared between subgroups (Dzongkhags, gender, age groups, education level, and indigenous knowledge level) using the Chi-square test (Table 3). WEF consumption varied significantly between Trashigang and Trashiyangtse Dzongkhag (*p* < 0.05). Trashigang Dzongkhag had significantly more WEF consumers when compared to that in Trashiyangtse Dzongkhag. No statistically significant difference was found between the WEF consumption of women and men (*p* > 0.05). Similarly, education level also did not significantly affect WEF consumption. There was a significant association between age and WEF consumption (*p* < 0.05). Those between the ages of 40 and 50 were more likely to consume WEFs than the younger and elderly populations. Likewise, indigenous knowledge level was significantly correlated to WEF consumption (*p* < 0.05). In this study, indigenous knowledge was indicated by the number of species listed by the respondents. We considered respondents who cited more species as more knowledgeable than the others. The prevalence of WEF consumption was high for those who cited more species when compared with those who listed fewer species (*p* < 0.05).

**Table 3:** A comparison of WEF consumption among socio-demographic factors

Category	Total respondents	Number of respondents who consumed WEF	Proportions of respondents who consumed WEF	Chi^2^ value	*df*	*p*-value
**District**				7.63	1	**0.0058**
Trashigang	54	30	55.6			
Trashiyangtse	43	11	25.6			
**Gender**				1.47	1	0.226
Male	51	25	49.0			
Female	46	16	34.8			
**Age group (years)**						
20–30	15	4	26.7	12.14	4	**0.016**
31–40	23	8	34.8			
41–50	20	15	75			
51–60	16	7	43.8			
> 60	23	7	30.4			
**Education level**				6.31	2	0.097
Primary	17	3	17.6			
Secondary	7	3	42.9			
Illiterate	73	35	47.9			
**Number of WEF species listed**				14.1	4	0.0071
7–9	7	3	42.9			
10–12	41	11	26.8			
13–15	28	11	39.3			
15–18	16	12	75			
> 18	5	4	80			

*p-value* <0.05 represented in bold indicates a significant difference

**Fig. 3:**
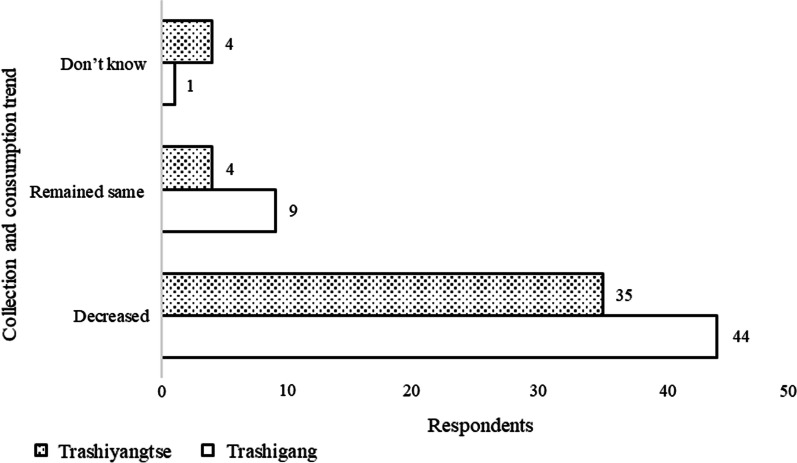
Respondents’ perception of the consumption trend of WEFs

The top 5 most consumed WEFs in the Dzongkhags were *Juglans regia*, *Myrica esculenta*, *Rubus ellipticus*, *Zanthoxylum armatum,* and *Phyllanthus emblica*. While 79 (81%) of respondents believed WEF consumption has decreased compared to that in the past, 13 (13.4%) perceived the trend remained the same, and 5 (5.14%) were unaware of the change in consumption trend (Fig. 3). The introduction of improved varieties, increased accessibility to improved varieties in the market, lower demand for WEFs in the market, changes in the food preferences, and lack of knowledge to identify species were the reasons cited by the respondents for the decreased consumption of WEFs. According to the respondents’ perception, out of 52 species, 85% were moderately and rarely available, while 15% were abundantly found.

### Indigenous knowledge holder

The average number of WEFs listed was compared between the subgroups of demographic factors using one-way ANOVA to determine the indigenous knowledge among different demographic characteristics (Table 4). Indigenous knowledge about WEFs varied significantly between the two Dzongkhags (*p* < 0.05). Trashigang had significantly higher indigenous knowledge compared to that in Trashiyangtse Dzongkhag. No statistically significant difference was observed between indigenous knowledge among men and women (*p* > 0.05). There was a significant association between age and indigenous knowledge (*p* < 0.05). Tukey’s test (post hoc test) was performed to compare the multiple interactions among five different age groups. Those aged 41–50 were likely to have more knowledge when compared to the age groups 20–30 and > 60. No significant difference was found in the indigenous knowledge and the education level of the respondents (*p* > 0.05).

**Table 4:** Comparison of subgroups of respondents on their indigenous knowledge about WEF

Category	Number of respondents	Average no of WEF listed	*F* value	*p* value
**District**			43.5	**0.0000000031**
Trashigang	54	14.5		
Trashiyangtse	43	11.4		
**Gender**			0.27	0.604
Male	51	13.2		
Female	46	13.0		
**Age group (years)**			2.99	**0.023**
20–30	15	11.7		
31–40	23	13.1		
41–50	20	15.1		
51–60	16	13.7		
> 60	23	12.0		
**Education level**			0.6744	0.512
Primary	17	13.8		
Secondary	7	12.9		
Illiterate	73	13.0		

*p-value* <0.05 represented in bold indicates a significant difference

## Discussion

### Diversity of WEF species and uses

In this ethnobotanical survey, we recorded 52 WEFs species from 35 botanical families in Trashigang and Trashiyangtse Dzongkhag, which is a larger number of species compared to that recorded in a previous study in Bhutan, in which 32 species of wild vegetables were reported from Tsirang Dzongkhag [26]. However, this is less when compared to studies conducted in Dagana and Trashiyangtse Dzongkhag, which reported 241 and 165 species of wild edibles [27, 37]. Similarly, a study conducted by Matsushima et al. [38] identified 172 wild edible species in Bhutan. A possible explanation for these differences could be the inclusion of all wild edibles like wild vegetables, wild fruits, cane, mushroom, and orchid, while the present study focused only on the WEFs. The number of wild edibles recorded in the present study is similar to that found in other regions of Asia, such as Pakistan [39], Indonesia [40], Western Himalaya [41], and Ethiopian countries [42, 43].

More WEFs were collected from the forest habitat compared to the surrounding fields. A similar finding was also reported by Regassa et al. [44] in Ethiopia. This result aligns with the term “wild,” which is more generally associated with unmanaged environments. The majority of the WEFs were collected during summer and autumn compared to those during winter and spring due to favorable climatic conditions for fruit setting and maturity, which is consistent with studies in Nepal, Pakistan, and Yunnan [21, 39, 45]. Rosaceae represented the greatest number of species which is in line with studies in Pakistan and India [39, 46].

WEFs were mostly consumed as both raw and cooked/processed, as most of them were used after drying or fermenting into wine. For example, fruits of *Docynia indica* and *Pyrus pashia* were consumed raw as well as dried. This result differs from studies in India and Estonia where WEFs were mostly consumed raw [46, 47]. Contrarily, species like *Dioscorea bulbifera*, *Colocasia esculenta,* and the flowers of *Oroxylum indicum* were cooked before consumption. WEF species such as *Rubus ellipticus*, *Docynia indica,* and *Juglans regia* were relatively common and familiar to the respondents and were extensively listed in both the Dzongkhags. Similarly, several past studies in Nepal, India, Laos, and Myanmar commonly reported *Colocasia esculenta* and *Phyllanthus emblica* for food and medicinal purposes [21, 23, 48, 49].

The ethnobotanical information showed that WEFs have multiple uses in addition to food, with more citations for their use as a raw material for furniture and construction, which is comparable with what was reported in Ethiopia [50], where the people highly exploited the species with multiple uses. Similar uses of WEFs among different communities in the two Dzongkhags indicated the existence of common traditional uses across different cultures and geographical areas, which is consistent with the reports of past studies in Ethiopia and Nepal [4, 21]. However, a higher citation for medicinal use of WEFs in Trashigang Dzongkhag might be due to some of the respondents being local healers and lay monks who commonly use wild edibles to treat local people. Species such as *Ficus auriculata* and *Ficus semicordata* were reported for fodder use as in Nepal [51]. The fruit species, *Catunaregam spinosa,* which is commonly called mountain pomegranate has been reported for its high medicinal value in other parts of South Asia [52, 53] but in the present study, the respondents mentioned only the food use of this species. This could be explained by the lower abundance of this species or limited knowledge on medicinal uses in the study area which could have restricted their use to consumption.

The medicinal use of WEFs generally included traditional remedies to treat common illnesses such as cough, dermal issues like skin irritation, pimples, and dandruff, which correspond to the results of a study in Nepal [21]. In this study, one plant species was cited for multiple health purposes; *Terminalia bellirica* was cited concerning six health uses: to treat cough, sore throat, diarrhea, ingestion, constipation, and asthma. However, medicinal use of the species was one of the least cited uses by the respondents, probably due to the accessibility of modern health facilities such as BHUs in each geog which is similar to that reported by Weckmüller et al. [54]. Similarly, *Zanthoxylum armatum* was reported to be the most commonly collected and consumed spices, as was the case in Yunnan, China [55]. Likewise, Yangtse geog was popular for its traditional paper made from the bark of *Daphne bholua,* which is used for painting and writing religious scripts. A similar finding was reported in Arunachal Pradesh, whose climatic conditions and religion are similar to that in Bhutan [56].

### WEF consumption

The present study demonstrated that the respondents mostly collected WEFs for self-consumption, with only a few species being sold in the local market for income generation, probably owing to the lack of or low market value for the WEFs [55]. Barely 9% of the respondents sold the WEFs, including the fruits of *Zanthoxylum armatum*, *Mangifera sylvatica,* and *Juglans regia*, to the local market for income generation. Despite 100% citations for food use by the respondents, the consumption of WEFs has decreased. Our observation found that the primary reasons for decreased consumption of WEFs were: (1) the introduction of improved varieties, (2) accessibility to improved varieties in the market, (3) less demand for WEFs in the market, (4) change in food preferences, and (5) lack of knowledge on identification of WEF species. These reasons are interrelated, as the introduction of improved varieties may have improved the accessibility to improved fruit varieties in the market, leading to decreased demand for WEFs in the market. Accordingly, Aryal et al. [41] also reported the negligence of traditional food due to changing food habits, taste, and availability of readymade foods in Western Himalaya. In addition to being easier to manage, the improved varieties are widely perceived as having better quality than WEFs. As WEFs grow in less ideal conditions, they are often smaller and produce fewer fruits that are less juicy and more seeded compared to the improved varieties [57]. Hence, the shift in preference from the wild to the improved varieties is understandable.

The present study found that middle-aged people, 41–50 years old, consumed more WEFs than the younger and older populations. These people are generally energetic in the villages, working closely with nature. Moreover, this age group has more indigenous knowledge, resulting in higher consumption. However, this finding contrasts with Nepal and Pakistan where young boys involved in cattle herding in the forest consumed more WEFs [21, 39]. Likewise, the Trashigang residents consumed more WEFs than the residents of Trashiyangtse. This unequal distribution in consumption might be because of the difference in accessibility and acceptability of WEFs among the two Dzongkhags, which is in line with the findings of Bvenura & Sivakumar [58]. The WEFs in Trashiyangtse may be located extremely far away from the village, where people had to walk very long distances, affecting their consumption. Moreover, the result showed that only 26% of the respondents in Trashiyangtse had consumed WEFs in the last twelve months, indicating their dependence on improved varieties. Indigenous knowledge was significantly associated with WEF consumption which corresponds to the findings of Reyes-Garcia et al. [59]. Generally, people consume WEFs when they know the fruit is edible or has some health benefits. Contrary to the studies in Ethiopia and Indonesia, there was no significant association of WEF consumption with gender and education level [43, 60].

### Indigenous knowledge pattern

In line with other studies [21, 61], this study showed that indigenous knowledge of WEFs differed significantly between the Dzongkhags, with the respondents from Trashigang having more knowledge compared to those from Trashiyangtse. An average citation of 14.5 and 11.4 WEF species in Trashigang and Trashiyangtse, respectively, justifies the predominance of high indigenous knowledge in Trashigang Dzongkhag. Local healers and lay monks would have contributed to the higher level of indigenous knowledge in Trashigang Dzongkhag. Notably, age groups had a significant association with indigenous knowledge of WEFs. In this regard, we found high indigenous knowledge among middle-aged people in their 40s and 50s compared to younger and older age groups, which are consistent with studies done in Pakistan and Nepal [39, 62]. However, it contradicts the findings of Uprety et al. [21] where younger people were more knowledgeable than the older population, and some studies in China where the oldest generation had more traditional knowledge than others [55, 63]. Based on our field observation, there are three possible explanations for this tendency: firstly, people in their 40’s and 50’s were more knowledgeable due to first-hand experience; secondly, the less knowledge in younger generations, particularly from 20 to 30 years, would likely stem from their low interest in WEFs, and less exposure to the wild environment since the majority of the youngsters spend more time at schools or in town nowadays; thirdly, the declining knowledge exhibited by the senior citizens could be because they have less direct involvement in the forest.

In line with some studies in China [55, 64], the association between gender and indigenous knowledge was not statistically significant since people worked closely with nature irrespective of their gender. Nonetheless, the result contrasts with the findings in Ethiopia, Brazil, and Italy [43, 44, 65, 66] where women reported more wild edibles than men. On the contrary, Kang et al. [67] concluded that men were more knowledgeable in Central China. Similarly, studies from Nepal and Argentina also reported that men identified more fruit species than women [62, 68]. These three studies were conducted in communities with rich forest cover where it was always the men who ventured further into the forest. Likewise, this study also found no association between indigenous knowledge and the education level of the respondents. Generally, indigenous knowledge is transferred orally from parents to children requiring no academic qualification, which is consistent with the findings of Mengistu & Hager [69]. However, this result contrasts with the findings in Ethiopia, where literates possessed more indigenous knowledge [43, 44] while illiterates were more knowledgeable in China [64].

### Implications for promotion and conservation of WEFs

The present study found that WEF consumption has decreased compared to the past, resulting in the extinction of wild food culture and its associated indigenous knowledge. Thus, it is important to focus on promoting these neglected species before the culture of wild food consumption disappears. WEFs have a high potential to enhance food security and income generation in the remote areas of Bhutan owing to their high nutrient content and multiple uses. Hence, it is imperative to create awareness of the nutritional and other diverse uses of these species in the region. Regardless of its inferior quality and taste, the value addition of WEFs is reported to yield high returns to the farmers and increase the keeping quality [13]. In the study area, people hardly processed WEFs to make them value-added except for a few conventional practices, including drying and pickle making for consumption, owing to their limited skills in agro-processing and value addition. Thus, training programs on agro-processing and value addition are essential to diversify products and increase profit to the farmers [70]. Simultaneously, integrating wild-plant-related knowledge in the school curriculum would familiarize the youths with these important wild species and their associated indigenous knowledge.

This study found that 85% of the species were rarely and moderately found in the region, indicating the possible declining diversity of some species, which is perceived to be caused by deforestation, climate change, and overharvesting. Similar findings of decreasing availability of the species were reported in Nepal and Ethiopia [21, 43]. Hence, the future agroforestry agenda should prioritize the conservation and domestication of these rarely available species. Owing to their hardy nature and better adaptation to harsh climate than the improved varieties [58], and their resistance to drought and natural disasters such as fire [71], these wild species are suitable for planting in slide-prone areas. In addition, some WEF species like *Ardisia macrocarpa*, *Cornus capitata,* and other evergreen or deciduous trees with beautiful flowers and fruits also add an additional aesthetic value to landscapes and highways.

This study has a few limitations. Firstly, the duration of fieldwork was short and included only the individual surveys. Consequently, a logical follow-up would include participatory and focus group discussions. Secondly, the lack of marketing surveys as the WEF species were hardly sold in the market for income generation. This study attempted to document species diversity and ethnobotanical uses of WEFs in eastern Bhutan. Although the survey was limited to only two Dzongkhags, we believe that the results sufficiently represent the species diversity and indigenous knowledge in the east but may not be necessarily pervasive to other regions in Bhutan. Therefore, replicating this research based on a case study in other regions is advisable to elucidate more comprehensive information on species diversity and indigenous knowledge associated with WEFs.

## Conclusion and recommendation

This paper is the first ethnobotanical study of WEFs in eastern Bhutan. While this study found a rich diversity of WEFs in two Dzongkhags in eastern Bhutan, only 42% of the respondents consumed WEFs in the last twelve months showing the decreasing trend in WEF consumption, especially among younger generations. Hence, there is a need to explore agro-processing and value addition to boost the consumption and income generation, as these neglected species have a high potential to enhance food security in the remote areas of the country. Moreover, the study found a decline in species availability, necessitating conservation measures and domestication. Thus, subsequent studies on potential WEF species having an aesthetic and nutritional value can promote and conserve the species. The study further revealed that younger generations have less indigenous knowledge than the elderly, recommending the need for WEF-related knowledge inclusion in the school curriculum.

## Data Availability

A structured, organized version of the data and the voucher numbers of the voucher specimens will be available from the first author upon reasonable request.

## Abbreviations

WEF: Wild edible fruit
ARDC: Agriculture Research and Development Centre
Nu: Ngultrum (Bhutanese currency)
NBC: National Biodiversity Centre
ARDSC: Agriculture Research and Development Sub-Centre
masl: Meters above sea level
BHU: Basic Health Unit
JICA: Japan International Cooperation Agency
HRDP: Horticulture Research and Development Project

## Footnotes

1 An open-source tool for data collection and analysis. It is accessible at https://www.kobotoolbox.org/.
